# A Cross-Sectional Survey Assessing the Factors Influencing Dentists’ Decisions on Post-Endodontic Prosthetic Crown Restoration

**DOI:** 10.3390/jcm14113632

**Published:** 2025-05-22

**Authors:** Alexandru Gliga, Carlo Gaeta, Federico Foschi, Simone Grandini, Jose Aranguren, Xavier-Fructuos Ruiz, Adriano Azaripour, Mihai Săndulescu, Cezar Tiberiu Diaconu, Dana Bodnar, Marina Imre

**Affiliations:** 1 Department of Operative Dentistry, Faculty of Dental Medicine, “Carol Davia” University of Medicine and Pharmacy, 050474 Bucharest, Romania; dana21bodnar@gmail.com; 2 Unit of Endodontics, Department of Medical Biotechnologies, Periodontology, Restorative and Paediatric Dentistry, University of Siena, 53100 Siena, Italy; simogr@gmail.com; 3 Unit of Endodontology, Department of Restorative Dentistry, UCL Eastman Dental Institute, University College London, London WC1E 6DE, UK; 4 Department of Endodontics, Rey Juan Carlos University, 28032 Madrid, Spain; josearanguren@hotmail.com; 5 Department of Restorative Dentistry and Endodontics, Universitat Internacional de Catalunya, 08195 Barcelona, Spain; xavier.ruiz@uic.es; 6 Department of Oral and Maxillofacial Surgery, Plastic Surgery, University Medical Centre of the Johannes Gutenberg-University Mainz, 55131 Mainz, Germany; 7 Department of Implant Prosthetic Therapy, Faculty of Dental Medicine, “Carol Davila” University of Medicine and Pharmacy, 050474 Bucharest, Romania; mihai.sandulescu@umfcd.ro; 8 Faculty of Dental Medicine, Department of Endodontics, University of Medicine and Pharmacy of Craiova, 200349 Craiova, Romania; diaconu_czr@yahoo.com; 9 Department of Complete Denture, Faculty of Dental Medicine, “Carol Davia” University of Medicine and Pharmacy, 050474 Bucharest, Romania; melim.marina@gmail.com

**Keywords:** clinical decision-making, endodontic therapy, prosthetic restoration, restorative dentistry, Romanian dentists, treatment outcomes

## Abstract

Interdisciplinary decision-making significantly influences both the therapeutic potential and clinical outcomes, shaping clinical attitudes and management strategies. As the integration between endodontic and restorative-prosthetic considerations becomes increasingly prevalent, it is essential to understand how different dental specialists, particularly general dental practitioners, prosthodontists and endodontists, approach clinical decision-making and collaborate to optimize patient care. **Objectives**: This study aims to identify practice disparities in post-endodontic crown placement to inform national policy reforms, including standardised timing protocols and interdisciplinary referral criteria. **Methods**: A structured questionnaire was distributed to dentists practicing in Romania, yielding 238 collected responses. **Results**: Substantial variability was found in clinical approaches: diagnostic imaging preferences indicated frequent use of periapical radiography (83.49%) and CBCT (53.67%). Over 70% expressed high confidence in CBCT’s diagnostic precision, significantly higher than periapical radiography (Wilcoxon Signed-Rank test, *p* < 0.00001). A statistically significant majority (69.3%, binomial test, *p* < 0.001) preferred delaying definitive crown placement until radiographic healing of periapical lesions. Logistic regression analysis showed endodontists were significantly less likely to choose invasive treatments compared to other specialists (*p* = 0.027). Although clinicians widely recognize the significance of prosthetic planning, its early integration into the overall treatment strategy has been inconsistent. **Conclusions**: This study points out the necessity for standardised guidelines that clearly integrate prosthetic planning into endodontic decision-making, enhancing predictability and tooth preservation.

## 1. Introduction

Endodontic therapy is fundamental within dentistry, aiming to preserve teeth affected by pulpal and periapical diseases [1]. Although successful outcomes of endodontic treatment depend heavily on the quality of root canal procedures, adequate restoration, whether direct or indirect, equally plays a critical role [2]. Endodontically treated teeth present structural vulnerabilities, requiring strategic prosthetic planning to ensure their long-term survival [3,4]. Despite the existing scientific evidence supporting the importance of the prosthetic phase of the treatment, such considerations frequently remain overlooked during initial treatment planning, potentially resulting in overtreatment or insufficient structural protection [5,6].

Clinical decision-making in endodontics is inherently complex, involving precise diagnostics, therapeutic selection and careful prognosis evaluation. Dentists historically rely on periapical radiography to guide treatment decisions; however, the limitations of this bi-dimensional imaging method, including overlapping anatomical structures and poor sensitivity in differential diagnostics, have been well documented [7,8]. Recent advances in diagnostic imaging, notably cone-beam computed tomography (CBCT), offer enhanced accuracy, significantly influencing clinical decisions and improving diagnostic confidence [9,10].

The integration of prosthetic considerations in the endodontic clinical decision-making is vital, directly influencing prognosis, functional rehabilitation and overall patient satisfaction [11,12]. Nonetheless, an element of subjectivity still persists when assessing prosthetic factors with regard to endodontic treatment planning. Dentists’ varying levels of expertise across endodontics, prosthodontics and restorative dentistry may lead to substantial variability in clinical approaches [13], decision-making processes [14,15,16] and ultimately, treatment outcomes [17]. Furthermore, potential drawbacks associated with multidisciplinary approaches have been highlighted by Slavicek et al. (2013) [18].

This study aims to bridge this knowledge gap by assessing Romanian dentists’ decision-making behaviours concerning prosthetic considerations in endodontic practice. By identifying disparities in clinical approaches to post-endodontic crown placement, this research provides essential evidence to guide national policy reforms, including the development of standardised timing protocols and clearly defined interdisciplinary referral criteria. Specifically, the study evaluates the dentists’ diagnostic imaging preferences, factors influencing restorative decision-making and the perceived role of prosthetic planning in clinical success. Given the evident variability in clinical practice, findings from this research could inform future guidelines, educational frameworks and training programs, ultimately supporting a more integrated, evidence-based approach in endodontic and prosthetic dentistry.

## 2. Materials and Methods

A cross-sectional, electronic survey was conducted among practicing Romanian dentists to evaluate attitudes, perspectives and clinical practices related to prosthetic considerations and endodontic therapy. Ethical approval was obtained from the Carol Davila University Ethics Committee. Participation in the survey was voluntary, anonymous and an informed consent was obtained electronically, being included in the Google Forms document.

This survey is part of a larger multicentric study, intended to gather comparative data from five countries, with this research focusing exclusively on responses from Romania. The structured questionnaire, developed specifically for this study by the primary investigators and registered with the Open Science Framework (https://doi.org/10.17605/OSF.IO/HA78D, accessed on 27 February 2025), included demographic data (age, gender, professional experience, practice location and dental specialty), diagnostic imaging preferences, clinical decision-making scenarios, interdisciplinary collaboration practices, attitudes toward conservative and minimally invasive approaches, and restorative planning considerations. The questionnaire was carefully designed to minimize biased responses, employing psychologically nuanced questions that presented varying clinical scenarios. While all items pertained to the same core subject, their formulation aimed to engage respondents’ decision-making processes as comprehensively as possible. The survey was reviewed by three endodontists and two prosthodontists, achieving a content validity index (CVI) greater than 0.7, thus confirming its suitability and relevance for the intended research objectives. A pilot testing was run prior to launching the survey by an international research team (*n* = 12) to ensure clarity, relevance and comprehensibility; minor adjustments were required following this pilot phase. Twenty dentists participated in a second pilot test to assess internal consistency, with Cronbach’s alpha confirming a reliability coefficient (α) greater than 0.7 for the decision-making items.

Dentists were identified and recruited through professional networks, including WhatsApp, Facebook, Instagram groups, academic institutions and dental associations. The inclusion criteria were clearly defined as follows: all dentists needed to hold a valid license, actively practice dentistry in Romania and be fluent in Romanian to be able to comprehend the survey questions. The survey was administered online via Google Forms (Google LLC, Mountain View, CA, USA), and multiple reminders via text messages and phone calls were employed to maximize participation rates.

A total of 238 complete responses were collected over a four-week data collection period, out of which, 20 were students and did not meet the inclusion criteria. Consequently, the final number of responses taken into consideration for analysis was 218, meeting the minimum sample size required to ensure statistical relevance, calculated based on the total number of dental practitioners in Romania. This sample size was calculated with the Id Survey Sample Size Calculator (https://www.idsurvey.com/en/sample-size-calculator/, accessed on 1 February 2025), allowing a margin of error of approximately ±6.6% at a 95% confidence level. No missing data handling was necessary, as all collected responses were complete.

Data analysis involved descriptive statistics to summarize demographic characteristics and survey response distributions. Inferential analyses, including Chi-square tests for categorical variables and logistic regression analyses, were employed to examine associations between dentists’ perceptions, clinical decision-making behaviours and demographic characteristics. Statistical significance was set at *p* < 0.05 and analyses were performed using statistical software (SPSS, version 28.0; IBM Corp., Armonk, NY, USA). Additional statistical validation and subgroup analyses are planned to enrich the results for future publications.

## 3. Results

**Figure 1:**
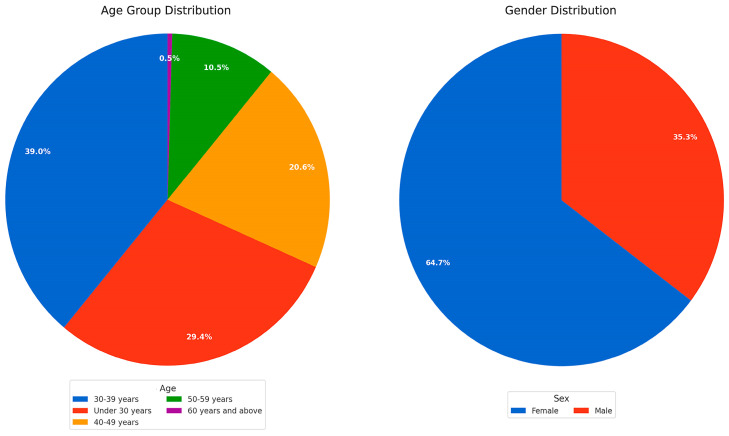
The first pie chart describes the age group distribution of the survey participants, while the second pie chart reveals the gender distribution of the respondents.

**Figure 2:**
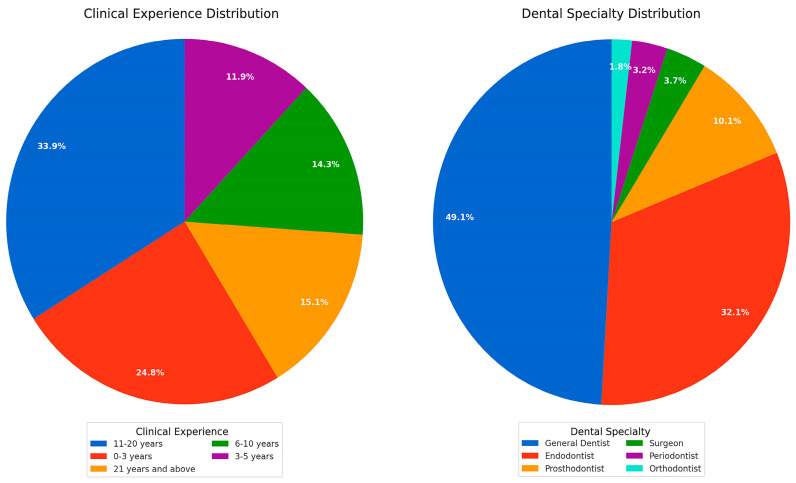
Pie chart 1 explains the clinical experience distribution of the survey participants. Pie chart 2 shows the dental specialty distribution of the respondents.

This survey collected data covering 218 evenly-distributed responses with a range of clinical experience and an appropriate distribution of respondents by gender, representative for the gender distribution in the dental profession, according to the national employment statistics [19], thus offering a coherent picture of how Romanian dentists think and guide their approach (Figure 1 and Figure 2).

### 3.1. Diagnostic Imaging Preferences and Perception

**Figure 3:**
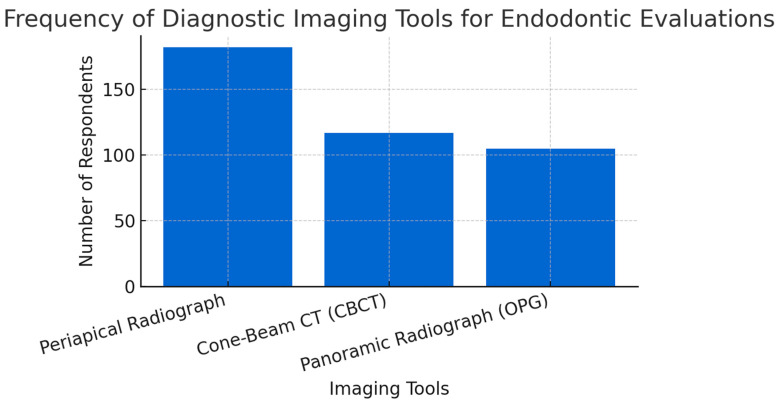
The frequency of diagnostic imaging tools for endodontic evaluations.

Dentists reported frequent use of diagnostic imaging modalities, predominantly periapical radiography (83.49%), followed by cone-beam computed tomography (CBCT, 53.67%) and lastly, panoramic radiography (OPG, 48.17%) when considering endodontic evaluation and treatment planning (Figure 3).

More than 70% of the dentists reported a high confidence level in the accuracy of CBCT investigations for differentiating between periapical cysts and granulomas (χ^2^ = 93.70, *p* < 0.001). Subgrouping was performed to identify any association between the dental specialty and diagnostic perception, but no statistical significance results were found.

**Figure 4:**
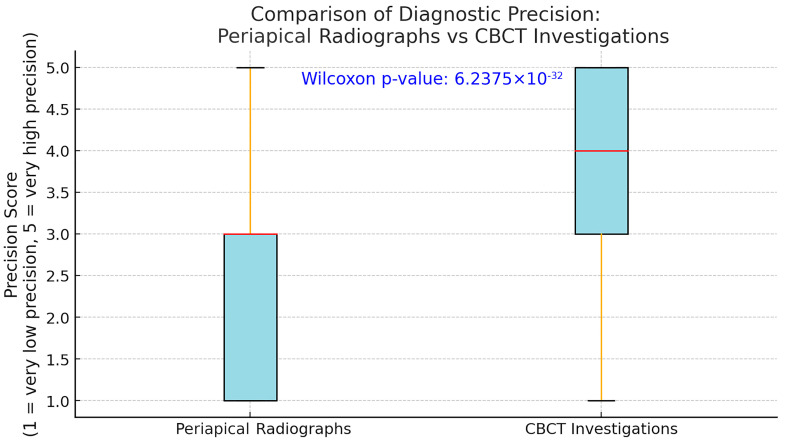
Comparison of diagnostic precision: periapical radiographs vs. CBCT investigations, illustrating dentists’ perceived diagnostic precision comparing periapical radiographs (left, box shaded in light blue) and Cone-Beam Computed Tomography (CBCT; right, box shaded in light blue) for differentiating between periapical cysts and granulomas. Each box represents the interquartile range (25th to 75th percentile), with the horizontal red line indicating the median precision score. Whiskers (orange lines) extend to the minimum and maximum values, showing variability beyond the quartiles. Precision was rated on a Likert scale from 1 (very low precision) to 5 (very high precision). The highly significant Wilcoxon Signed-Rank test result (*p* = 6.2375 × 10^−32^, shown in blue at the top) indicates a statistically robust difference between the two methods. Specifically, dentists perceived the diagnostic precision of CBCT (median score approximately 4) significantly higher than that of periapical radiographs (median score approximately 3). This statistical significance underscores clinicians’ greater trust and confidence in CBCT as a diagnostic tool for accurately identifying cystic lesions compared to traditional periapical radiographs.

A Wilcoxon Signed-Rank test was conducted to analyse dentists’ perception of the capacity of periapical X-ray for differential diagnosis between periapical cyst and granuloma compared to cone-beam computed tomography. The results show a strong statistical significance (*p* < 0.00001), concluding that there is a higher trust in CBCT accuracy in detecting cystic lesions compared to periapical X-rays (Figure 4).

### 3.2. The Influence of CBCT Diagnostic Precision on Clinical Decision-Making

#### Impact of CBCT Diagnostic Precision on Treatment Choices

**Figure 5:**
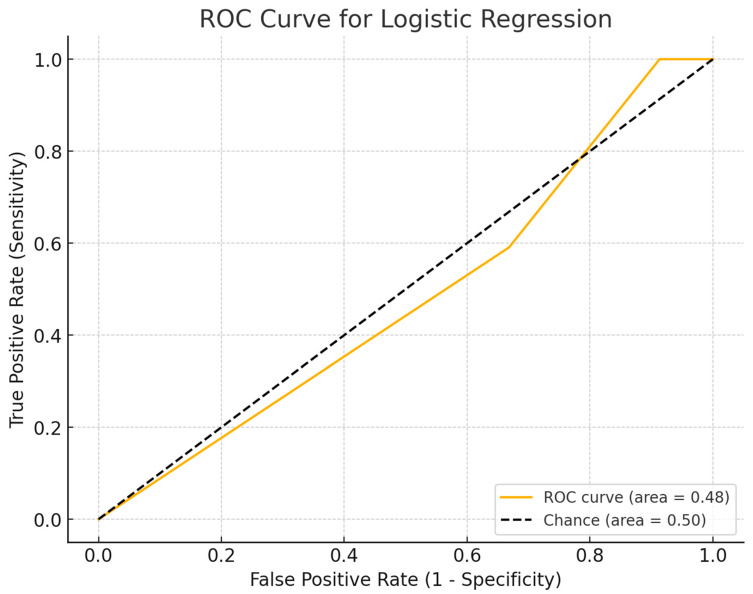
ROC (receiver operating characteristic) curve assessing the predictive accuracy of a logistic regression model developed to determine dentists’ decision-making regarding invasive versus conservative therapeutic approaches based on perceived CBCT diagnostic precision. The ROC curve is represented by the solid orange line, illustrating the model’s true positive rate (Sensitivity) versus the false positive rate (1—Specificity) at various threshold settings. The diagonal dashed black line represents the reference line of chance (Area Under Curve [AUC] = 0.50), indicating the expected performance of a completely random classifier. The calculated Area Under the Curve (AUC) for the logistic regression model (0.48, shown in orange within the legend) indicates performance slightly below chance, suggesting that the logistic regression model based solely on dentists’ perceived precision of CBCT has very limited predictive value in distinguishing between invasive and conservative clinical decisions. Thus, this finding highlights the need for including additional variables or predictors to enhance clinical decision-making models.

A logistic regression analysis was performed to evaluate whether dentists that considered CBCT a good diagnostic tool to differentiate cysts from granuloma leaned towards a more invasive approach in cases where a cyst was confirmed. The regression model showed a predictive accuracy of 89.91%, indicating strong predictive validity. However, dentists’ perceived accuracy of CBCT imaging did not significantly influence their preference for invasive treatments such as immediate apicoectomy, extraction or referral to oral surgery (*p* = 0.850), even if the clinical scenario also presented with a separated instrument. Dentists across specialties consistently preferred conservative treatment options, such as endodontic retreatment with monitoring or referral to an endodontic specialist, irrespective of their trust in CBCT (Figure 5).

**Figure 6:**
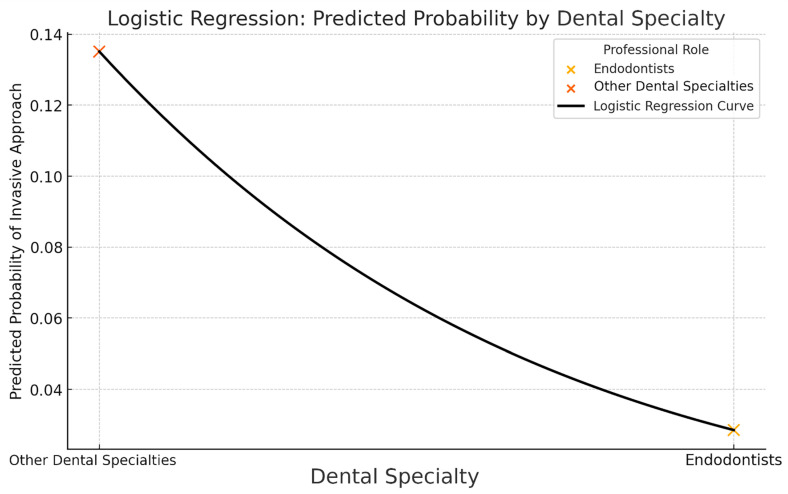
Logistic regression: predicted probability by dental specialty. Logistic regression curve illustrating the predicted probability of choosing an invasive therapeutic approach according to dental specialty. The solid black logistic regression curve demonstrates the model’s predicted probability gradient between other dental specialties (left) and endodontists (right). The probability of choosing an invasive approach is notably higher for other dental specialties (orange “X” marker on the upper-left) compared to endodontists (light orange “X” marker on the lower-right). These visual markers indicate the model’s predicted probability at each group, clearly highlighting a lower predicted likelihood of invasive clinical decisions among endodontists compared to their peers in other dental specialties. This logistic regression model suggests specialty-specific differences in clinical decision-making, highlighting endodontists’ greater inclination toward conservative therapeutic approaches.

The same statistical analysis was performed for the subgroups “Endodontists” and “Other Specialties” with a predictive accuracy of 89.91%. The result shows that endodontists are five times less likely to choose an invasive approach compared to the other dental specialties. This effect was statistically significant *p* = 0.027 (Figure 6).

### 3.3. Effect of CBCT Precision on Decision to Postpone Definitive Restorations

In a separate logistic regression, the influence of perceived CBCT diagnostic accuracy on decisions regarding the postponement of definitive restoration provision in teeth that underwent endodontic treatment was dictated by the presence of a cyst-like lesion still in the process of healing. The results demonstrated a limited positive trend (coefficient = 0.135, *p* = 0.213), however this trend did not reach levels of statistical significance.

Subgroup analysis was performed to enhance the statistical interpretation; however, the results did not reach statistical significance (*p* = 0.073). While no definitive conclusions could be drawn, the data suggested a trend indicating that endodontists tended to delay definitive restorations less frequently than general dentists.

### 3.4. Clinical Decisions Influenced by a Hypothetical Availability of a Minimally Invasive Histological Diagnostic Tool

**Figure 7:**
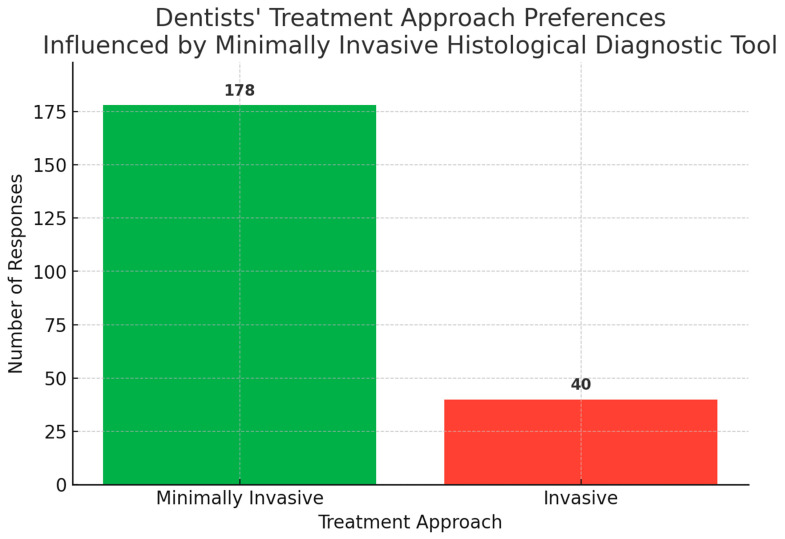
Dentists’ treatment approach preferences influenced by minimally invasive histological diagnostic tool.

A Chi-square analysis (Chi^2^ = 47.145) assessed dentists’ preferences for invasive versus minimally invasive treatment approaches when provided with a certified histological diagnosis prior to their clinical approach. The analysis revealed a highly significant preference (Chi^2^ = 47.145, *p* < 0.00001) towards conservative decisions (81.65%, *n* = 178) compared to invasive approaches (18.35%, *n* = 40). No statistically significant differences in decision-making were observed between endodontists and dentists from other specialties (Chi^2^ = 0.2432, *p* = 0.6219), suggesting broad consensus across dental specialties in preferring minimally invasive treatments when advanced diagnostics are available (Figure 7).

### 3.5. Rationale and Time Span for Final Crown Restoration in Cases with Ongoing Periapical Healing

**Figure 8:**
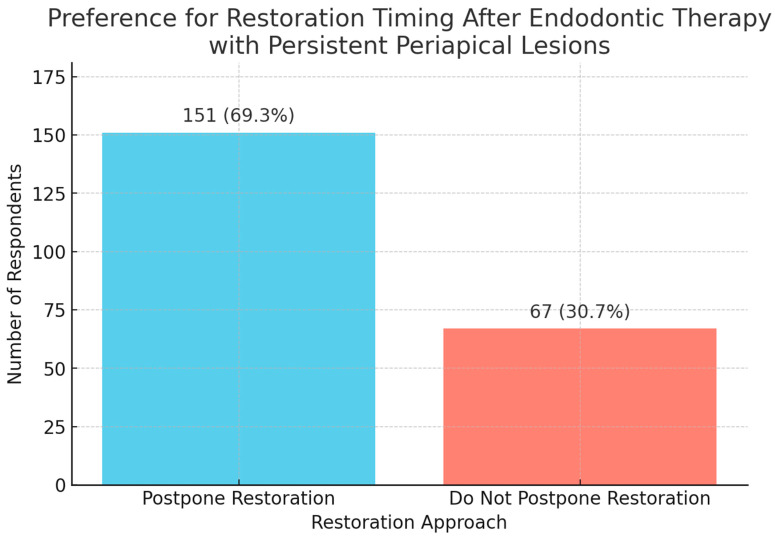
Preference for restoration timing after endodontic therapy with persistent periapical lesions.

A descriptive analysis (binomial test) explored the decision to postpone the definitive restoration in cases with periapical lesions still undergoing monitoring. A strong and statistically significant preference was found among respondents (69.3% vs. 30.7%) for postponing the definitive restoration until complete radiographic healing was found (*p*-value = 1.26 × 10^−8^). After subgrouping, a notable difference in decision-making patterns between endodontists and other dental specialists regarding restorative timing in cases with periapical lesions still under monitoring was observed. Endodontists showed no statistically significant preference (*p*-value = 0.1196) between postponing and not postponing restoration. Other dental specialties exhibited a strong and statistically significant preference (*p*-value = 7.46 × 10^−9^) for postponing the definitive restoration until complete radiographic healing was confirmed (Figure 8).

**Figure 9:**
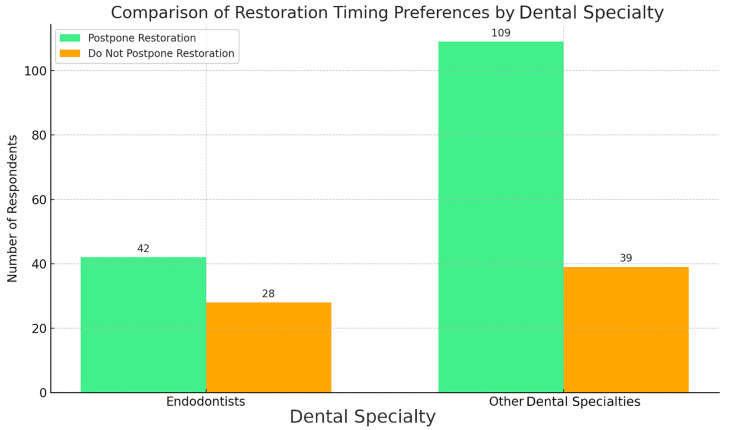
Comparison of restoration timing preferences by dental specialty.

A Chi-Square Test (Chi^2^ = 3.54) was conducted to compare the preferences between endodontists and other dental specialties regarding postponing the definitive restoration provision, but no statistically significant difference was found (*p* = 0.0598) (Figure 9).

### 3.6. Factors Influencing Decision-Making for Final Restoration and Treatment Options

#### 3.6.1. Restorative Approach for Endodontically Treated Teeth

“What factors influence your decision when selecting the most appropriate restoration for an endodontically treated tooth? (Please rank them in order of importance.)”

A Friedman test was first conducted to analyse the question-response effect. A highly significant *p*-value (*p* = 2.19 × 10^−41^, *p* < 0.001) indicates that there are statistically significant differences in how dentists ranked six factors influencing restorative decisions for endodontically treated teeth.

For further investigation, a Wilcoxon Signed-Rank Test and Bonferroni Correction were conducted, comparing each factor against every other factor, calculating *p*-values for each comparison and applying a stringent correction to keep conclusions conservative and reliable (Table 1).

**Figure 10:**
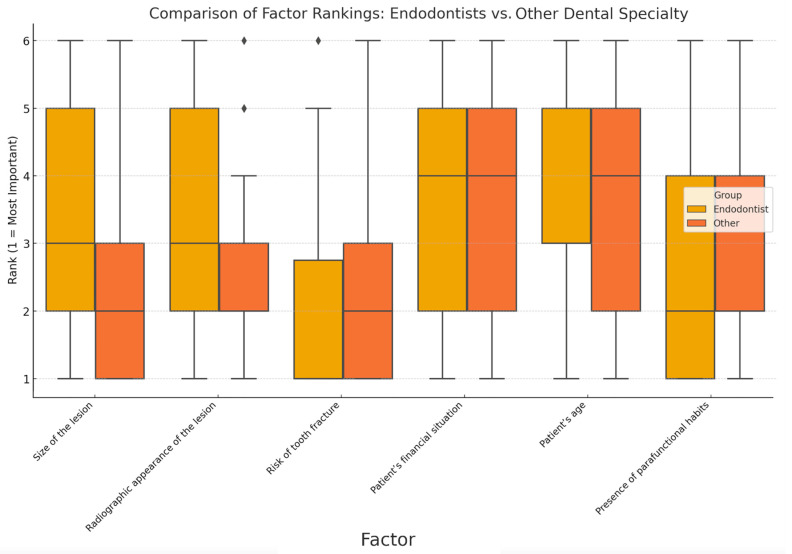
Comparison of factor rankings: endodontists vs. other dental specialty. The boxplots compare the rankings of factors influencing clinical decisions for tooth restoration between endodontists (yellow boxes) and other dental specialists (orange boxes). Each factor was ranked on a scale from 1 (most important) to 6 (least important). Boxes represent the interquartile range (IQR, 25th–75th percentile), with horizontal lines within each box indicating the median rank. Vertical lines (whiskers) denote the range of ranks observed, while the rhombi (⧫) above the boxplots indicate statistical outliers, representing individual responses significantly differing from group consensus. Size of the lesion and radiographic appearance of the lesion demonstrate notably higher importance (lower median ranks) for endodontists (yellow boxes) compared to other specialists, suggesting greater emphasis by endodontists on imaging-based diagnostic criteria. Factors like patient’s financial situation and patient’s age show relatively similar rankings between both groups, indicated by overlapping yellow and orange boxes, reflecting common agreement on these factors’ moderate importance. The factor presence of parafunctional habits (e.g., bruxism) appears to have slightly higher importance for other dental specialists (orange boxes) relative to endodontists, as indicated by a lower median rank among the former. This comparison highlights specific differences in clinical priorities and considerations between endodontists and their peers.

Using the Mann–Whitney U Test, significant differences between endodontists and other dental specialists were identified regarding factors influencing their final restorative treatment choices (Figure 10):

Endodontists tend to rank “Size of the lesion” and “Radiographic appearance of the lesion” significantly lower than other specialists.

For “Risk of tooth fracture”, both groups rated it highly (medians = 1 and 2), with no statistically significant difference.

Patient-related factors (age and financial situation) were similarly perceived across professions.

Endodontists attributed lower significance to the following factors: “Size of the lesion” and “Radiographic appearance of the lesion”, which aligns with the significant Mann–Whitney U results.

#### 3.6.2. To Preserve or to Extract?

“What are the most important factors influencing your decision between performing endodontic retreatment followed by prosthetic restoration or opting for extraction? (Please select all that apply and rank them in order of importance.)”

Additionally, a Friedman test (132.54) assessed the relative importance of factors influencing decisions between tooth extraction and endodontic retreatment followed by definitive restoration. Results demonstrated highly significant differences in factor prioritization (1.85 × 10^−25^). For further investigation, a Wilcoxon Signed-Rank Test and Bonferroni Correction were conducted, comparing each factor against every other factor, calculating *p*-values for each comparison and applying a stringent correction to keep conclusions conservative and reliable (Table 2).

**Figure 11:**
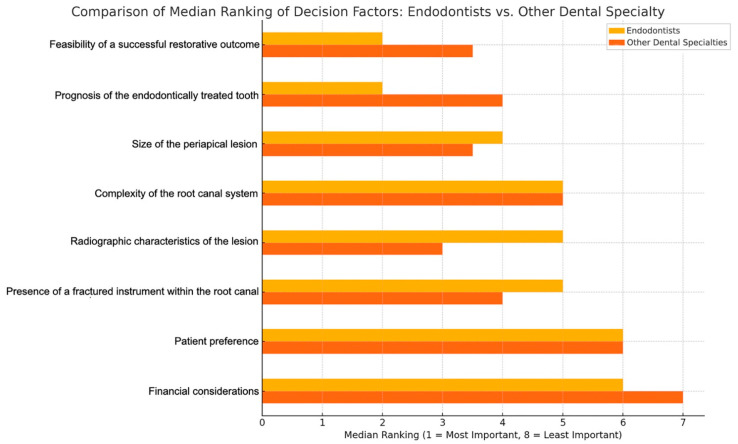
Horizontal bar chart comparing the median rankings of factors influencing clinical decision-making for tooth extraction versus endodontic retreatment with prosthetic restoration between endodontists (yellow bars) and other dental specialties (orange bars). Each factor was ranked from 1 (most important) to 8 (least important), with shorter bars indicating greater importance attributed by the respective group. Radiographic characteristics of the lesion, complexity of the root canal system and presence of a fractured instrument within the root canal show notably shorter yellow bars, highlighting that endodontists prioritize these factors significantly more than other dental specialists. This indicates endodontists’ greater emphasis on specific clinical and technical factors related directly to endodontic treatments. Conversely, financial considerations and patient preference display longer yellow bars and shorter orange bars, suggesting these aspects carry relatively greater importance for other dental specialists compared to endodontists. Factors such as size of the periapical lesion and prognosis of the endodontically treated tooth exhibit similar bar lengths for both groups, indicating agreement across dental specialists regarding their moderate importance. This clear visual differentiation emphasizes the distinct priorities and perspectives of endodontists compared to other dental professionals.

To statistically evaluate differences in the relative importance assigned by endodontists compared to non-endodontic dental specialists regarding various factors influencing clinical decision-making between tooth extraction and endodontic retreatment with subsequent definitive restoration, a Mann–Whitney U Test (two-tailed, α = 0.05) was used (Figure 11).

Endodontists considered the “Feasibility of a successful restorative outcomes” as the most critical factor (median rank = 2.0, closer to 1 indicates higher importance).

Non-endodontic specialists prioritized “Radiographic characteristics of lesions” as their most important factor (median rank = 3.0).

These analyses clearly demonstrate differentiated clinical reasoning patterns between endodontists and other dental specialists regarding restorative treatment decisions, influenced by their specific clinical focus and professional expertise. The observed association between specialty training and adherence to clinical guidelines highlights the importance of implementing targeted continuing education programmes. Poor radiographic interpretation skills, reported by 70% of surveyed dentists, could potentially explain the delays in definitive crown placement indicated by 30% of respondents, highlighting a critical area for targeted professional training.

## 4. Discussion

The results of this study offer very interesting insights with a novel perspective for future research because of the robust variation and discrepancies found. Although 70% of the dentists reported a high confidence level in the capacity of CBCT to provide accurate data about the implied histological status of a periapical lesion, many publications clearly report that unless serial sectioning histopathology investigation confirms the diagnosis, no routinely used imaging tool can currently achieve this [7,10,20,21,22]. Overestimating the capacity of a diagnostic tool can lead to misdiagnosis, thus raising the risk of misguided therapies, so there is a need to increase dentists’ awareness of the limitations of their devices and to suppress the inaccurate marketing claims provided by manufacturers or distributors within the industry.

Conversely, it is an interesting finding that dentists who consider CBCT to correctly diagnose the presence of cysts avoid the surgical approach, even if a histological confirmation was hypothetically available. Such an approach can be justified for endodontists, who are five times less likely to perform invasive treatment and rely on more advanced techniques to deal with large lesion, as expected due to their specialized training. According to Faggion’s findings, cariology and restorative dentistry are reported as having the highest levels of available evidence, whereas endodontics and prosthodontics are ranked as less supported specialties [23]. Considering other specialties, this indicates that clinical decisions might also heavily rely on other factors such as clinician experience, patient-specific conditions or even treatment philosophy, beyond just diagnostic capabilities. This aligns with findings from Murdoch et al., which demonstrate that clinical decisions under uncertainty are influenced significantly by clinicians’ cognitive biases, training and heuristic reasoning, rather than purely by the diagnostic capabilities of available tools [24]. Moreover, this observation raises concerns regarding the decision-making process among respondents, suggesting potential biases influenced by a defensive attitude, likely triggered by awareness that the research questionnaire responses will undergo detailed analysis, or even possibly reflecting a preference to be perceived as practitioners aligned with the currently favoured minimally invasive clinical model, as per social desirability bias.

This perceptible trend towards conservative clinical management is underscored by further results indicating dentists’ strong belief that preserving tooth structure during endodontic procedures directly affects teeth longevity and long-term prosthetic success. A rational approach to preserving healthy tooth structure when performing cavity access, root canal preparation, management of endodontic complications and crown preparation is a crucial factor for avoiding errors and increasing the chances of achieving a favourable outcome [25,26,27,28,29]. Excessive dentine-sparing approaches may lead to an increased number of iatrogenic instances, especially for non-specialists.

The timing for placing the definitive restoration is always subject to analysis when there is an ongoing healing process; however, prolonged use of temporary restorations possesses risks, such as coronal leakage and tooth fracture, potentially compromising the long-term success of endodontically treated teeth [30,31]. Goodacre and Spolnik clearly emphasize that the timely placement of definitive restorations and careful tooth preparation considerations significantly influence the prognosis of endodontically treated teeth, supporting timely restorative interventions to mitigate risks associated with delayed restoration [32,33]. Moreover, using composite or amalgam build-ups as definitive restorations following root canal treatment significantly compromises tooth survival, more than doubling the risk of extraction [17]. Additionally, delaying crown placement beyond four months after treatment further increases this risk, nearly tripling it compared to crowns placed within four months [17]. This study clearly demonstrates that Romanian dentists frequently prefer provisional restorations and monitoring before proceeding to definitive treatments. This cautious behaviour may reflect not only a conservative philosophy, but potentially a defensive dentistry mindset, driven by practitioners’ fear of complaints or litigation, thus leading to delayed definitive restoration [34].

The ranking of factors influencing dentists’ treatment decision contrasts with previous findings of the survey, suggesting future avenues of investigation. Although the responses at the beginning of the survey suggested that lesion characteristics did not lead dentists to favour invasive treatment, the final section of the questionnaire shows that lesion size and radiographic features significantly impact clinicians’ decisions—both in choosing the restoration method and determining whether to preserve the tooth. A common concern among general dental practitioners, prosthodontists and endodontists is the risk of tooth fracture, which was rated by all as highly important.

The reported high level of interdisciplinary collaboration, particularly with prosthodontists, highlights dentists’ acknowledgment of the critical role played by a multidisciplinary approach when restoring structurally compromised endodontically treated teeth. Yet, the observed variability in collaboration practices underscores an evident need for clearer and evidence-based clinical guidelines. Limitations associated with multidisciplinary approaches have been discussed by Slavicek et al. (2013), emphasizing challenges such as communication barriers between specialties, stemming from unclear guidelines, divergent clinical opinions, team conflicts, workflow disruptions, as well as increased treatment costs for patients, reluctance among practitioners towards adopting new conceptual frameworks and the heightened effort and professional commitment required [18]. Consistent with previous recommendations in the literature, such guidelines could reduce variability and standardize practice patterns, ultimately benefiting patient outcomes and clinical efficiency [35]. Additionally, Alani et al. emphasize how specialty training, experience and clinical discussions substantially influence restorative treatment planning, further supporting the necessity for structured educational frameworks to harmonize clinical decision-making [13].

A comparison between these findings and current Romanian dental curricula, which includes minimal interdisciplinary training, suggests that deficiencies in radiographic interpretation and subsequent delays in definitive crown placement may stem from inadequate integration of endodontic–prosthodontic education. Therefore, a comprehensive revision of the curricula to incorporate greater interdisciplinary training appears necessary to address these educational gaps effectively. Furthermore, specific policy actions are recommended: revising curricula to explicitly include integrated endodontic and prosthodontic modules; introducing compulsory continuing professional development (CPD) programmes targeting clinical and diagnostic competencies; establishing formal accreditation standards to assess interdisciplinary skills through practical evaluations and Objective Structured Clinical Examinations (OSCEs) and fostering regular interdisciplinary collaboration within dental faculties through structured seminars and workshops. These targeted educational and regulatory measures have the potential to substantially improve clinical outcomes and elevate the overall standard of patient care.

The external validity of this study was limited by its geographically confined survey distribution, primarily capturing responses within a single national context. Additionally, a potential urban bias should be acknowledged, as rural practitioners may exhibit different decision-making patterns influenced by varying access to technology and clinical practice characteristics. Although statistically significant results were obtained, future research would benefit from expanding the survey to include practitioners from multiple countries and diverse geographic areas, thus ensuring a larger, more representative sample and enhancing the overall generalizability and comprehensiveness of the findings.

## 5. Conclusions

The variability and inconsistencies in prosthetic considerations and clinical approaches evident from our results highlight a critical need for well-defined, comprehensive guidelines, as suggested by previous consensus reports [35]. Standardised, evidence-based protocols can significantly reduce uncertainty and variability in clinical practice, thus improving decision-making consistency and enhancing treatment outcomes.

Actionable recommendations emerging from this research should focus on guideline development, professional training initiatives and interdisciplinary protocols. Specifically, the establishment of a national/international consensus-based guideline addressing optimal crown placement timing (such as recommending definitive crown placement within four weeks for premolars) would provide clinicians with clear, actionable guidance. Additionally, targeted professional training initiatives, including practical workshops dedicated to enhancing periapical radiographic interpretation skills, are essential to addressing identified diagnostic shortcomings. To further support interdisciplinary clinical practice, the implementation of standardised electronic health record (EHR) templates that facilitate shared endodontic–prosthodontic treatment planning is strongly recommended.

In conclusion, these findings reinforce the necessity for comprehensive, evidence-based guidelines and ongoing interdisciplinary professional education. Systematically integrating prosthetic considerations into endodontic therapy, targeted diagnostic training and structured clinical protocols can significantly enhance clinical outcomes, treatment predictability and ultimately, tooth survival, which represents one of the main patient-centred outcome.

## Figures and Tables

**Table 1:** Pairwise Wilcoxon Signed-Rank Test results.

	Factor 1	Factor 2	Statistic	p-Value	Significant (Bonferroni Corrected)
1	Size of the lesion	Radiographic appearance of the lesion	3875.0	1.0	FALSE
2	Size of the lesion	Risk of tooth fracture	4544.5	0.00026579870299298943	TRUE
3	Size of the lesion	Patient’s financial situation	3998.0	2.5333148609749084 × 10^−7^	TRUE
4	Size of the lesion	Patient’s age	3362.5	5.054794611413946 × 10^−9^	TRUE
5	Size of the lesion	Presence of parafunctional habits	6825.0	1.0	FALSE
6	Radiographic appearance of the lesion	Risk of tooth fracture	3755.0	1.5845899673177555 × 10^−6^	TRUE
7	Radiographic appearance of the lesion	Patient’s financial situation	4405.0	4.067956797586387 × 10^−7^	TRUE
8	Radiographic appearance of the lesion	Patient’s age	3606.5	8.554452406846989 × 10^−10^	TRUE
9	Radiographic appearance of the lesion	Presence of parafunctional habits	6491.5	1.0	FALSE
10	Risk of tooth fracture	Patient’s financial situation	2111.5	3.74610556252516 × 10^−17^	TRUE
11	Risk of tooth fracture	Patient’s age	2195.0	1.0288567882695264 × 10^−18^	TRUE
12	Risk of tooth fracture	Presence of parafunctional habits	2321.5	2.9566901945733574 × 10^−9^	TRUE
13	Patient’s financial situation	Patient’s age	6646.0	1.0	FALSE
14	Patient’s financial situation	Presence of parafunctional habits	3955.0	7.713597598065507 × 10^−9^	TRUE
15	Patient’s age	Presence of parafunctional habits	2728.5	2.3366778678366123 × 10^−12^	TRUE

**Table 2:** Pairwise Wilcoxon Signed-Rank Test results.

	Factor 1	Factor 2	Statistic	*p*-Value	Significant (Bonferroni Corrected)
1	Complexity of the root canal system	Size of the periapical lesion	3055.5	0.00942309800732801	FALSE
2	Complexity of the root canal system	Radiographic characteristics of the lesion	3426.0	0.09090837381321469	FALSE
3	Complexity of the root canal system	Presence of a fractured instrument within the root canal	3535.0	0.15406392799101443	FALSE
4	Complexity of the root canal system	Feasibility of a successful restorative outcome	2441.5	5.68631249962933 × 10^−5^	TRUE
5	Complexity of the root canal system	Patient preference	3204.0	0.027403705329801967	FALSE
6	Complexity of the root canal system	Prognosis of the endodontically treated tooth	3366.5	0.06915552397079221	FALSE
7	Complexity of the root canal system	Financial considerations	2481.0	8.565811606481425 × 10^−5^	TRUE
8	Size of the periapical lesion	Radiographic characteristics of the lesion	3627.5	0.21991907946614342	FALSE
9	Size of the periapical lesion	Presence of a fractured instrument within the root canal	2585.0	0.0002052044869450844	TRUE
10	Size of the periapical lesion	Feasibility of a successful restorative outcome	3048.0	0.009710989800152451	FALSE
11	Size of the periapical lesion	Patient preference	2344.0	2.0237440865496785 × 10^−5^	TRUE
12	Size of the periapical lesion	Prognosis of the endodontically treated tooth	4004.0	0.7672471076754539	FALSE
13	Size of the periapical lesion	Financial considerations	1775.0	1.9902665000139928 × 10^−8^	TRUE
14	Radiographic characteristics of the lesion	Presence of a fractured instrument within the root canal	2832.0	0.0017001905490849063	TRUE
15	Radiographic characteristics of the lesion	Feasibility of a successful restorative outcome	2658.0	0.0004160384080060211	TRUE
16	Radiographic characteristics of the lesion	Patient preference	2569.5	0.00019124779035497368	TRUE
17	Radiographic characteristics of the lesion	Prognosis of the endodontically treated tooth	3632.5	0.23595436286445182	FALSE
18	Radiographic characteristics of the lesion	Financial considerations	1956.5	2.1317018227860162 × 10^−7^	TRUE
19	Presence of a fractured instrument within the root canal	Feasibility of a successful restorative outcome	1684.0	4.735348646975183 × 10^−9^	TRUE
20	Presence of a fractured instrument within the root canal	Patient preference	3323.5	0.05375150498927233	FALSE
21	Presence of a fractured instrument within the root canal	Prognosis of the endodontically treated tooth	2720.0	0.00075594097039443	TRUE
22	Presence of a fractured instrument within the root canal	Financial considerations	2346.5	2.0581099499104012 × 10^−5^	TRUE
23	Feasibility of a successful restorative outcome	Patient preference	1235.5	4.6189821806569014 × 10^−12^	TRUE
24	Feasibility of a successful restorative outcome	Prognosis of the endodontically treated tooth	2875.5	0.0024075203911395108	FALSE
25	Feasibility of a successful restorative outcome	Financial considerations	834.5	3.451978225000826 × 10^−15^	TRUE
26	Patient preference	Prognosis of the endodontically treated tooth	1969.0	2.182473830534536 × 10^−7^	TRUE
27	Patient preference	Financial considerations	2629.0	0.0002795080718228816	TRUE
28	Prognosis of the endodontically treated tooth	Financial considerations	1182.5	1.823849199508511 × 10^−12^	TRUE

## Data Availability

The data that support the findings of this study are available from the corresponding author, (G.A.), upon reasonable request.
